# Autophagy deficiency protects against ocular hypertension and neurodegeneration in experimental and spontaneous glaucoma mouse models

**DOI:** 10.1038/s41419-023-06086-3

**Published:** 2023-08-24

**Authors:** Angela Dixon, Myoung Sup Shim, April Nettesheim, Aislyn Coyne, Chien-Chia Su, Haiyan Gong, Paloma B. Liton

**Affiliations:** 1 Department of Ophthalmology & Pathology, Duke University, Durham, NC 27705 USA; 2 Department of Ophthalmology, Boston University School of Medicine, Boston, MA 02118 USA

**Keywords:** Diseases, Neurological disorders, Neurodegeneration

## Abstract

Glaucoma is a group of diseases that leads to chronic degeneration of retinal ganglion cell (RGC) axons and progressive loss of RGCs, resulting in vision loss. While aging and elevated intraocular pressure (IOP) have been identified as the main contributing factors to glaucoma, the molecular mechanisms and signaling pathways triggering RGC death and axonal degeneration are not fully understood. Previous studies in our laboratory found that overactivation of autophagy in DBA/2J::GFP-LC3 mice led to RGC death and optic nerve degeneration with glaucomatous IOP elevation. We found similar findings in aging GFP-LC3 mice subjected to chronic IOP elevation. Here, we further investigated the impact of autophagy deficiency on autophagy-deficient DBA/2J-*Atg4b*^ko^ and DBA/2J-*Atg4b*^+/−^ mice, generated in our laboratory via CRISPR/Cas9 technology; as well as in *Atg4b*^ko^ mice subjected to the experimental TGFβ2 chronic ocular hypertensive model. Our data shows that, in contrast to DBA/2J and DBA/2J-*Atg4b*^+/−^ littermates, DBA/2J-*Atg4b*^ko^ mice do not develop glaucomatous IOP elevation. Atg4b deficiency also protected against glaucomatous IOP elevation in the experimental TGFβ2 chronic ocular hypertensive model. *Atg4* deletion did not compromise RGC or optic nerve survival in *Atg4b*^ko^ mice. Moreover, our results indicate a protective role of autophagy deficiency against RGC death and ON atrophy in the hypertensive DBA/2J-*Atg4b*^+/−^ mice. Together, our data suggests a pathogenic role of autophagy activation in ocular hypertension and glaucoma.

## Introduction

Glaucoma is a group of diseases, which is characterized by the chronic degeneration of retinal ganglion cell (RGC) axons and progressive loss of RGCs, resulting in visual field defects and vision loss. Glaucoma is the leading cause of permanent blindness worldwide. Elevated intraocular pressure (IOP) and aging are the best-well known factors contributing to the onset and progression of glaucoma [1]. Current therapeutic treatments for glaucoma are targeted at lowering IOP, but they cannot rescue RGC loss. One major limitation to the development of novel neuroprotective treatments is that the pathobiology of glaucoma is still not fully understood [2]. Elevation in IOP is known to lead to a mechanical lesion of RGC axons within the lamina cribrosa of the optic nerve (ON) head that results in Bax-dependent apoptotic RGC death [3]. On the cellular and molecular level, this glaucomatous damage is characterized by impaired axonal transport, mitochondrial dysfunction, astrocytic malfunctioning, local inflammatory reactions, glutamate toxicity, and increased TNF-alpha [4]. However, the exact molecular mechanisms and signaling pathways triggering RGC death and axonal degeneration have not been fully identified.

Autophagy is a lysosomal degradative process, which plays a central role in cellular homeostasis by eliminating damaged organelles and proteins. In addition to maintaining cellular and tissue homeostasis, autophagy is regarded as a survival pathway, involved in stress-induced adaptation [5]. Dysfunction of autophagy has been associated with a growing number of human diseases, in particular age-related diseases, as well as to several neurodegenerative disorders. Paradoxically, despite having primarily a pro-survival role, excessive or uncontrolled levels of autophagy can induce cell death, especially in the neural tissue [6–8]. This dual role of autophagy is associated with the intricate cross-talk between the apoptotic and the autophagic signaling pathways [9, 10].

Autophagy in glaucoma has been investigated by independent laboratories using different experimental models [11–22]. While all of the studies agree that autophagy is activated in RGC in response to injury or elevated IOP, there is conflicting evidence regarding its pro-survival or pro-cell death role. These controversial results are explained, at least partly, by the use of different model systems (traumatic injury vs acute elevation in IOP vs chronic elevation in IOP), species and examination times; the use of non-specific pharmacological modulators of autophagy rather than genetic models; and frequent use of RGC death as the only readout with no evaluation of the effect or status of autophagy in RGC axons. In a previous work, our laboratory investigated the contribution of autophagy to ocular hypertension and glaucoma in the spontaneous glaucoma model DBA/2J [12]. DBA/2J is a genetic mouse model of glaucomatous neurodegeneration. At around 6 months of age, DBA/2J mice develop eye abnormalities (i.e iris atrophy and pigment dispersion) that lead to glaucomatous elevation in IOP and subsequent RGCs and ON axons loss [23]. For these studies, we generated the transgenic mice DBA/2J::GFP-LC3 mice, which ubiquitously express the autophagosome marker LC3 fused to GFP. Very interestingly, we found that expression of the GFP-LC3 transgene resulted in higher elevation in IOP and exacerbated RGC and axonal degeneration compared to the littermates DBA/2J controls. Furthermore, such exacerbated neurodegeneration was accompanied by higher presence of autophagic figures within degenerating axons [12]. Similar results were observed in aging GFP-LC3 mice subjected to chronic IOP elevation [15]. These results strongly suggested that overactivation of autophagy could be a potential cellular mechanism leading to RGC death and ON degeneration with glaucomatous IOP elevation.

Here, we followed up on these studies and further investigated the role of autophagy in glaucoma in autophagy-deficient DBA/2J-*Atg4b*^ko^ mice, which we generated in our laboratory, and in *Atg4b*^ko^ mice subjected to the transforming growth factor beta 2 (TGFβ2) chronic ocular hypertensive model. Our data shows that autophagy deficiency protects against glaucomatous IOP elevation, RGC death and axonal degeneration.

## Materials and methods

### Animal husbandry, genotyping and tissue collection

DBA/2J and C57BL/6J mice were purchased from Jackson Laboratories. *Atg4b*^ko^ mice were obtained by rederivation of cryopreserved sperm by in vitro fertilization in C57BL/6J females at Duke Transgenic and Knockout Mouse Core Facility. Cryopreserved sperm was obtained from Dr. Herbert Virgin’s lab at Washington University School of Medicine with permission from Dr. Carlos Lopez-Otin’s lab at the University of Oviedo (Spain), who originally generated the transgenic line [24]. Transgenic DBA/2J-*Atg4b*^ko^ mice were generated by deleting a ~ 8 kb region encompassing exons 4–8 in the *Atg4b* gene via CRISPR/Cas technology using Duke Transgenic and Knockout Mouse Core Services. Deletion of this region results in a frameshift mutation beginning in exon 9 and a premature STOP codon in exon 10 of *Atg4b*. Knockout strategy and sgRNAs sequences are included as Supplementary Fig. 1. PCR-based genotyping of digested tail genomic DNA was performed under standard conditions using the following primers: DBA/2J-Atg4b-1: ACTTTCCTCATCCTTACTGTCAGC; DBA/2J-Atg4b-2: CCCACACAATTTGTTGCTACTCAA; DBA/2J-Atg4b-3: GAAACCCTGACTAAGACAACTTCC. Zygosity was determined based on the amplification of a wildtype 650 bp product with primers 1 and 2; or a 250 bp transgenic product with primers 1 and 3. The presence of the Tyrp1^b^ allele in DBA/2J mice was confirmed by assaying a polymorphism creating a *TaqI* restriction-enzyme site in exon 4 within PCR products amplified from genomic DNA (TyrpF: CAGGAGCCTTCTTTCTCCCT; TyrpR: AAAGTGTCCCAGGGTATCG). The presence of the *Gpnmb*^R150X^ mutation was confirmed by assaying the *PvuII* restriction-enzyme site created by the mutation (GpnmbF: CTACAACTGGACTGCAGGGG; GpnmbR: AGCTCCATTTCTTCCATCCA). Zygosity of *Atg4b*^ko^ mice was determined based on the amplification of a wildtype 500 bp product or a 350 bp transgenic product with primers Atg4b-1, 2 and 3 (Atg4b-1: CCTCCAGCTCACTGAACTCC; Atg4b-2: CACGCCATACAGTCCTCTTCA; Atg4b-3: AAGTATATAGGCCTGGATGGTGCT). All mouse strains were maintained and bred as heterozygous. Homozygous and littermate wildtypes were used for experimental purposes. Animals were maintained under a 12-h light/dark cycle, fed a standard mouse diet, and provided with water *ad libitum*. Animal euthanasia was performed via CO_2_ asphyxiation, followed by bilateral thoracotomy, prior to immediate eye enucleation. Enucleated eyes were perfused with either 4% paraformaldehyde (PFA) for immunofluorescence or 2% glutaraldehyde/2%PFA in 1xPBS for electron microscopy as described earlier [12]. Tissues destined for western blot were immediately dissected and flash frozen on dry ice. All procedures were reviewed and approved by the Institutional Animal Care and Use Committee of Duke University and were performed in accordance with the ARVO Statement for the Use of Animals in Ophthalmic and Vision Research and the National Institutes of Health *Guide for the Care and Use of Laboratory Animals*.

### Intraocular pressure (IOP) measurements

IOP was measured using a Tonolab rebound in isoflurane-anesthetized mice. A series of six measurements per eye (OS and OD), taken within one minute post anesthesia, were collected and averaged to produce a single IOP value per eye for each measurement session. While under anesthesia, mouse body temperature was maintained at 38–40 °C by using Deltaphase isothermal pads (Braintee Scientific, Inc.). IOP was measured twice per month for aging studies and twice per week in the experimental TGFβ2 ocular hypertensive mouse model. Integral IOP (cumulative pressure received by each mouse on the entire duration of the experiment) was calculated using the Area Under the Curve (AUC) tool of GraphPad Prism 5 software and expressed as mmHg-day. Integral IOP difference (∆Integral IOP = Integral IOP_injected_-Integral IOP_uninjected_) was used as a measure of IOP exposure in the injected compared to the non-injected eye for each animal.

### Ex-vivo outflow facilities in mouse eyes

Eyes were enucleated within 5 min of death and kept in a warmed solution of 5.5 mM glucose in Dulbecco’s PBS with Calcium and Magnesium (DBG) until perfusion (15–20 min). Outflow facility measurements were conducted ex-vivo using the iPerfusion MK V system [25], as we previously described [15]. Briefly, eye cups were glued to platforms within heated baths filled with DBG and cannulated through the anterior chamber with a glass microneedle and a XYZ micromanipulators (World Precision Instruments, Sarasota, FL). An initial pressure of 8 mmHg for a period of 30 min was applied to allow acclimatization of the eyes to pressure and temperature environment. A nine-step post acclimatization protocol was conducted with applied pressures of 5.0, 6.5, 8.0, 9.5, 11.0, 12.5, 14.0, 15.5, 17.0 mmHg before returning to 8.0 mmHg. Outflow facility was calculated with the iPerfusion software.

### Experimental TGFβ2 ocular hypertensive mouse model

C57BL/6J mice (male, females) were unilaterally injected with a recombinant lentivirus expressing constitutively active human TGFβ2 (C226, C228S) under the CMV promoter (L-TGFβ2, VectorBuilder, Product ID: VB170816-1094fnw; Chicago, IL, USA). Empty lentivirus vector (L-Null, VectorBuilder, Product ID: VB191207-1096qrr; Chicago, IL, USA) was used as control. For this, animals were anesthetized with ketamine (100 mg/kg)/xylazine (10 mg/kg) cocktail. Before the injections, proparacaine HCl 0.5% (Akorn Inc., Lake Forest, IL, USA) anesthetic drops were applied on the cornea to ensure complete insensitivity. Viral suspensions (2 × 10^6^ TU/eyes, 2 μL bolus) were intravitreally injected in the pars plana region of the sclera via Hamilton glass micro-syringe and glass microneedles manufactured in-house by pulling glass capillary tubes (PN-3 Puller, Narishge Scientific Instrument Lab, Tokyo, Japan) and sharpened to a 20 degree bevel (BV-10, Sutter Instrument Company, Novato, CA). Mouse body temperature was maintained at 38–40 °C by using Deltaphase isothermal pads (Braintee Scientific, Inc.).

### Light and electron microscopy

Whole eye cups were post-fixed for 24 h in 2% PFA/2% glutaraldehyde in 1xPBS solution. Samples were then washed washed in 1xPBS and post-fixed for 90 min in 1% OsO4 in 1xPBS. Complete dehydration was achieved using an increasing ethanol gradient ending in two cycles of propylene oxide. Tissue was infiltrated by immersion in a 1:1 mixture of propylene oxide and Epon 812 resin under a vacuum for 4–10 h. Labeled molds were then heated at 68 degrees F for at least 8 h. Plastic embedded sections (0.5 μm) were stained with toluidine blue for light microcopy. For electron microscopy, sections were cut at 65 nm thick using a Leica EM CU7 and contrast stained with 2% uranyl acetate/4% lead citrate solution. Ultrathin sections were visualized on a JEM-1400 transmission electron microscope using an ORIUS (1000) CCD camera.

### Immunofluorescence on frozen tissue sections

Tissue samples were fixed in 4% PFA, cryopreserved, and sectioned as previously described [12]. For immunolabeling, sections were permeabilized with 0.5% Triton-X/PBS for 10 min at room temperature and incubated in 2% BSA/5% normal goat serum/0.1% Triton-X/PBS for 30 min at room temperature to block non-specific sites. Samples were then incubated overnight at 4 °C with primary antibodies diluted in a blocking solution, washed with 1 x PBS, and incubated for 1 h at room temperature in Alexa Fluor 568 Goat anti-Rabbit antibody (Thermo Fisher Scientific) diluted 1:1000 in serum-free blocking solution. Nuclei were counterstained with DAPI (1:1000 in PBS). Images were collected using a Nikon TE2000 confocal microscope. All tissues to be compared were immunostained at the same time, and images were captured the same day under the same laser settings to avoid interexperimental variability. The following antibodies were used in this study: anti-GFAP (AB53554 from Abcam), anti-cCASP3 (Asp175, Cell Signaling).

### Whole retinal flat mounts and retinal Ganglion cell (RGC) quantification

Retinas were fixed in 4% PFA, washed three times for ten min in 0.5% Triton-X/1X PBS at room temperature, and permeabilized overnight in 2% Triton-X/1X PBS at 4 ^o^C with agitation. Retinas were then incubated for three days at 4 ^o^C with anti-Brn3a (Santa Cruz Biotechnology, 1:750 in 2% Triton-X/2% Normal Donkey Serum/1X PBS), washed in 2% Triton-X/1X PBS and 0.5% Triton-X/1X PBS, and incubated at room temperature for 4 h in Alexa-Fluor Donkey anti-Goat 568 (Life Technologies, 1:500 dilution in 2% Triton-X/1X PBS). Tissues were counterstained in Hoechst (1:1000 in 0.5% Triton-X/1X PBS) for 30 min at room temperature, washed in 0.5% Triton-X/1X PBS, and mounted in aqueous mounting media (Fluormount G; Electron Microscopy Sciences). Retinal flat mounts were imaged with a EZ-C1.3.10 Nikon TE2000 confocal microscope (40× lens) (D-ECLIPSE C1, Nikon). A series of four images were taken at approximately equidistant points along peripheral circumference of the retina, and in the central region. DAPI-stained nuclei and Brna3-satined nuclei were quantified in a masked fashion by two different observers using Image J.

### Optic nerve axon count

ONs were fixed in 4% PFA, 1% glutaraldehyde in 1xPBS for 24 h. Samples were then washed, processed and embedded in the Embed-812 resin mixture. Blocks were sectioned on an ultramicrotome (LKB Ultratome V; Leica) using a glass knife. Cross sections were stained with 1% toluidine blue. Axon counts were obtained using the AxioVision imaging system (Zeiss). Image analysis consisted of RGB thresholding, followed by size and form factor exclusions. Approximately 40% of the total cross-sectional area for each ON was counted, and the results were extrapolated to the entire nerve for each mouse.

### Protein whole tissue lysate preparation & western blots

Dissected tissue was homogenized by manual grinding in cold RIPA buffer containing protease and phosphatase inhibitor cocktails (Thermo Scientific) and molecular grinding resin (G Biosciences). Lysates were subjected to three freeze/thaw cycles and clarified by centrifugation at 12,000 × *g* for 30 min at 4 °C. Protein concentration was determined with a protein assay kit (Micro BCA, Thermo Scientific). Protein lysates (3–5 μg) were separated by polyacrylamide SDS-PAGE gels [15% polyacrylamide for LC3 detection (NB100-2331 from Novus Biologicals) and 7% polyacrylamide for fibronectin (SC-8422 from Santa Cruz) and SQSTM1 (P0067 from MilliporeSigma)] and transferred to PVDF membranes (Bio-Rad). Membranes were blocked with 5% nonfat dry milk in 0.1% Tween-20/TBS and incubated overnight with primary antibodies. The bands were detected by incubation with a secondary antibody conjugated to horseradish peroxidase and chemiluminescence substrate (ECL; GE Healthcare and ECL2, Thermo Scientific). Blots were scanned and analyzed by densitometry using Image J. β-Actin (sc-69879) was used for loading control.

### Statistical analysis

All statistical analyses were performed using GraphPad Prism software. Data are presented as mean values ± SD using *T* test or Tukey post-hoc test. *P* < 0.05 was considered statistically significant. Sample size per each experiment is indicated within the text and/or in the corresponding figure legend and followed the recommendation in [26]. Studies were conducted in a masked approach.

## Results

### Generation of DBA/2J mice deficient in autophagy (DBA/2J–*Atg4b*^ko^)

To investigate the role of autophagy in neurodegeneration in glaucoma, we generated autophagy-deficient DBA/2J mice (DBA/2J-*Atg4b*^ko^) using CRISPR-Cas9 technology. For this, we deleted a ~ 8 kb region encompassing exons 4–8 in the *Atg4b* gene. This caused a frameshift mutation that resulted in a premature STOP codon in exon 10 (Supplementary Fig. 1A). Atg4b is an autophagy gene coding for the cysteine protease ATG4B, which is responsible for cleaving LC3 precursor (pro-LC3) into LC3-I, a key event for autophagosome formation. Deletion of Atg4b in DBA/2J was selected since *Atg4b*^ko^ mice are the only known mice deficient in autophagy that are not embryonically lethal [24]. Protein expression of Atg4b was not detected in retina, iridocorneal region or liver tissues from DBA/2J-*Atg4b*^ko^ mice (3-month-old) (Supplementary Fig. 1B). Higher basal pro-LC3 protein levels, resulting from defective cleavage were observed, similar to that described in *Atg4b*^ko^ mice [24]. In addition, DBA/2J-*Atg4b*^ko^ mice displayed the accumulation of SQSTM1, an autophagy receptor that is degraded by autophagy, thus confirming reduced basal autophagy activity in these mice. Transgenic DBA/2J-*Atg4b*^ko^ develop normally, but exhibit a significant deviation from Mendelian inheritance, suggesting potential prenatal lethality in this line. DBA/2J-*Atg4b*^ko^ and littermate controls, DBA/2J-*Atg4*^+/−^ and DBA/2J, were aged in situ up to 12 m.o. Unless otherwise indicated, all the quantification and analysis were conducted in tissues collected at this time-point.

### DBA/2J-*Atg4b*^ko^ mice do not develop elevated IOP

**Fig. 1:**
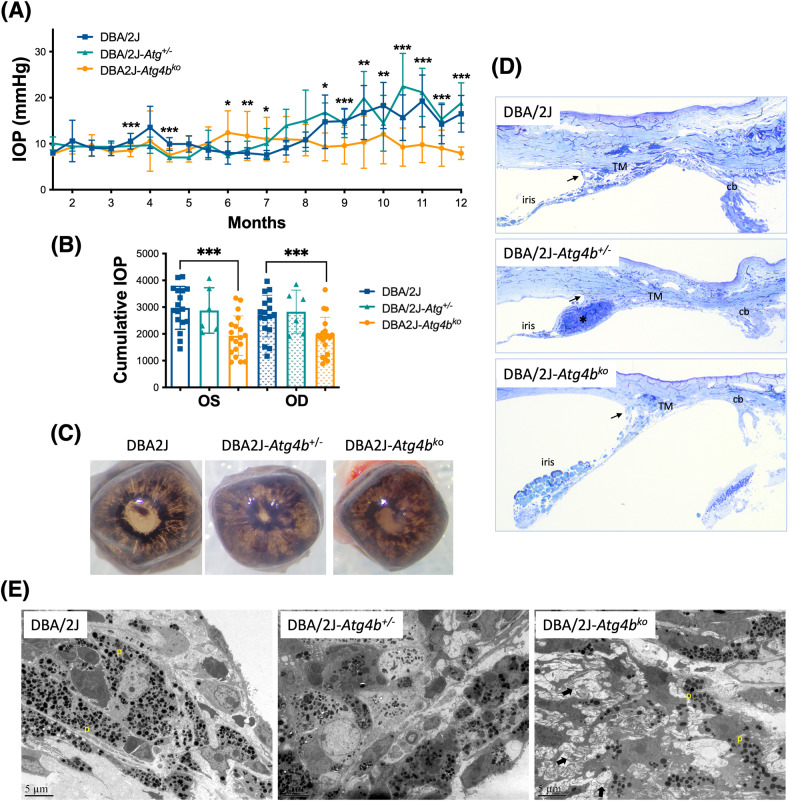
IOP and outflow pathway tissue morphology in autophagy deficient DBA/2J-*Atg4*^ko^ mice. **A** Mean IOP (mm Hg) monitored over time in DBA/2J, DBA/2J-*Atg4b*^+/−^ mice DBA/2J-*Atg4b*^ko^ mice. **B** IOP exposure throughout the duration of the experiment in the left (OS) and right (OD) eye. *p < 0.05, **p < 0.01, ***p < 0.001, ANOVA with Bonferroni post hoc test (DBA/2J *n* = 17, DBA/2J-*Atg4b*^+/−^ *n* = 6, DBA/2J-*Atg4b*^ko^ *n* = 19). **C** Macroscopic images of 4% PFA fixed eyes showing the incidence of pigmentary dispersion in WT and transgenic mice. **D** Toluidine blue-stained histological sections of the iridocorneal region showing anterior segments abnormalities. Closed angled is indicated by arrows. Images are representative of at least 6 animals per group. TM trabecular meshwork, cb ciliary body. **E** Ultrastructural images of the TM showing the accumulation of aberrant membranous structures (arrows) and diminished pigment particles (p) within TM cells.

We bi-monthly monitored IOP in DBA/2J-*Atg4b*^ko^ and littermate controls from 2 to 12 month of age. Figure 1A collects mean IOP measurements over time; Fig. 1B shows calculated IOP exposure in each eye (OS, OD) throughout the duration of the experiment (integral IOP). As expected, DBA/2J mice showed a gradual increase in IOP starting at around 7.5 months, peaking at ~20 mmHg at 11 months. Strikingly, while deletion of *Atg4b* in DBA/2J did not affect basal IOP, DBA/2J-*Atg4b*^ko^ mice failed to develop glaucomatous IOP elevation over time. In contrast, DBA/2J heterozygous for *Atg4b* (DBA/2J-*Atg4*^+/−^), behaved as littermate wt controls, showing similar IOP levels. Pigmentary dispersion was observed in both WT and transgenic mice (Fig. 1C), and no gross morphological difference was observed in the angle region in DBA/2J-*Atg4b*^ko^ compared to DBA/2J-*Atg4*^+/−^ and DBA/2J. DBA/2J-*Atg4b*^ko^ mice exhibited closed angle and anterior segment synechia, with various degrees of iris and ciliary body atrophy (Fig. 1D). At the ultrastructural level, DBA/2J-*Atg4b*^ko^ mice were characterized by the presence of membranous structures in the cells in the angle region and a potential decreased in pigment-laden cells (Fig. 1E), although no quantitative analyses were conducted to confirm that.

### ATG4B deficiency protects against RGC death in DBA/2J mice

**Fig. 2:**
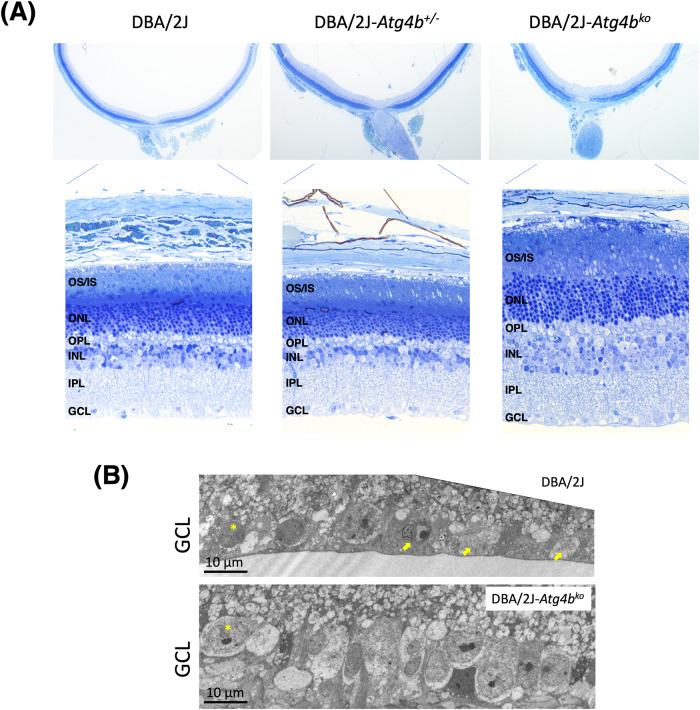
*Atg4b* deletion alters retinal morphology in DBA/2J mice. **A** Cross-sectional histological images showing changes in retinal morphology in DBA/2J-*Atg4b*^ko^ mice, including increased thickness of the INL and OS, and disorganization of the OS and IS (higher magnification shown in SM, Fig. 2). Images are representative of at least 6 animals per group. OS outer segment, IS inner segment, ONL outer nuclear layer, OPL outer plexiform layer, INL inner nuclear layer, IPL inner plexiform layers, GCL ganglion cell layer. **B** Ultrastructural images of the GCL showing the higher RGC density (asterisk, representative) and the absence of apoptotic and necrotic bodies (arrows) with deletion of *Atg4b*.

**Fig. 3:**
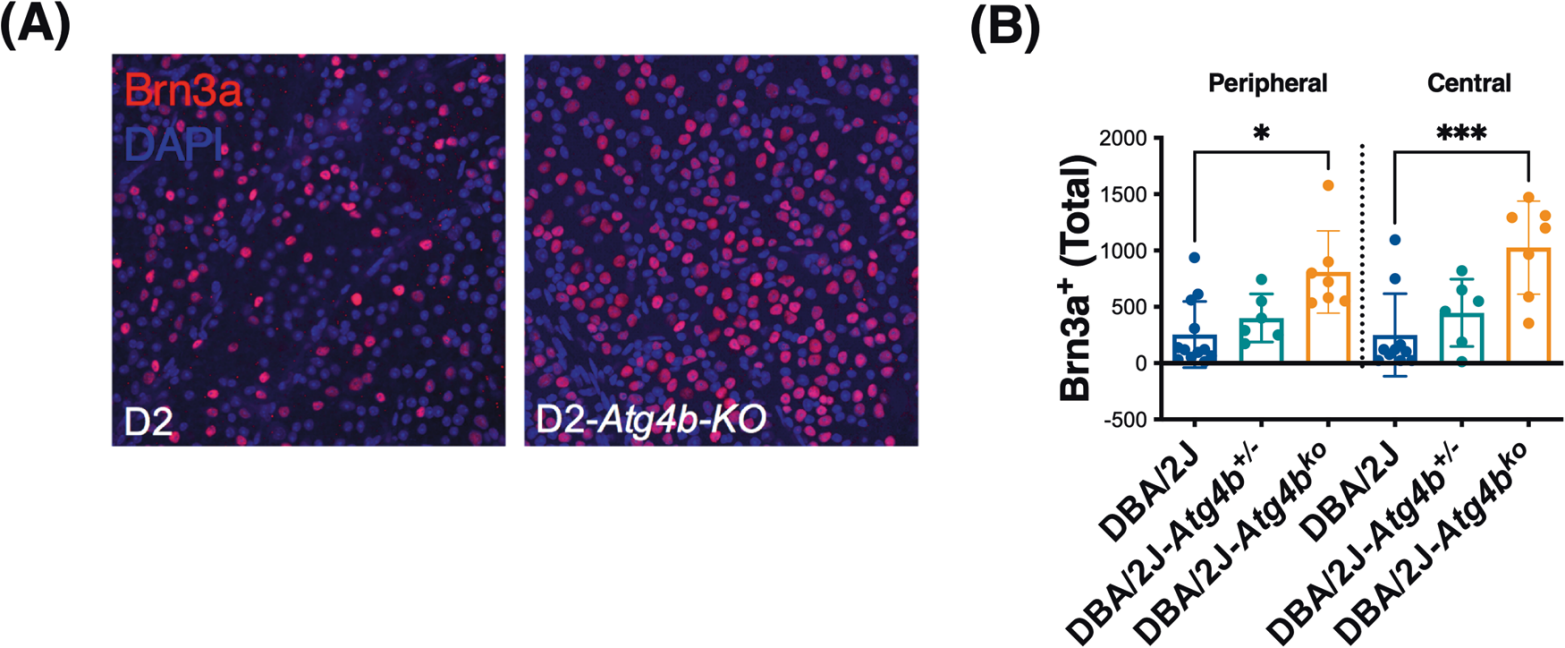
*Atg4b* deletion promotes RGC survival in DBA/2J mice. **A** Representative images from whole retinal flat mounts from DBA/2J and DBA/2J-*Atg4b*^ko^ mice (12 m.o.) stained with anti-Brn3a (red fluorescence). Blue represents DAPI nuclear staining. **B** Quantification of the number of Brn3a-positive cells in the peripheral and central retina in DBA/2J, DBA/2J-*Atg4b*^+/−^ and DBA/2J-*Atg4b*^ko^ mice (12 m.o.). Data are the means ± SD; **p < 0.01, Tukey test; (DBA/2J *n* = 12, DBA/2J-*Atg4b*^+/−^ *n* = 6, DBA/2J-*Atg4b*^ko^ *n* = 7).

Histological sections showed morphological differences in the retina tissue in DBA/2J-*Atg4b*^ko^ mice (Fig. 2A). Glaucomatous DBA/2J mice are characterized by thinning of the nerve fiber layer (NFL) and ganglion cell layer (GCL), with a single layer of scarce RGC. Thinning of the inner nuclear layer (INL), reduced to 3–4 layers of cells, is often observed [23, 27–29]. As depicted in the upper panels of Fig. 2A, the retinas of DBA/2J-*Atg4b*^ko^ exhibited increased thickness compared to Wt. Further examination at higher magnification revealed thicker INL, outer nuclear layer (ONL), inner segment (IS) and outer segments (OS). Notably, the IS and OS of DBA/2J-*Atg4b*^ko^ mice appeared structurally disorganized (Supplementary Fig. 2). These were also regionally observed in the heterozygous mice. No major ultrastructural differences in addition to higher RGC density was noted in the GCL and inner plexiform layer (IPL) in DBA/2J-*Atg4b*^ko^ compared to DBA/2J mice (Fig. 2B). To determine the effects of ATG4B deficiency on RGC death in glaucoma, we quantified RGCs in the peripheral and central region of the retina in whole retinal flat mounts immunostained with anti-BRN3A antibody. As seen in Fig. 3A, B, DBA/2J-*Atg4b*^ko^ mice showed significantly higher total RGC bodies compared to littermate controls. Partial RGC survival was also observed in DBA/2J-*Atg4b*^+/−^ mice.

### ATG4B deficiency prevents glaucomatous ON degeneration in DBA/2J mice

**Fig. 4:**
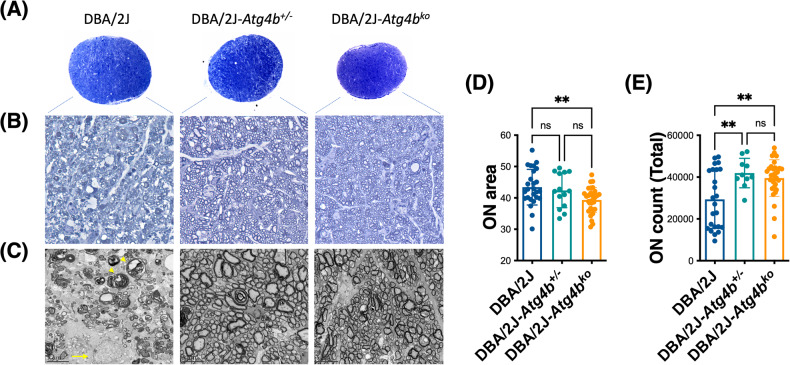
*Atg4b* deletion protects against ON degeneration in DBA/2J mice. **A** Low magnification, **B** high magnification and **C** electron micrographs of ON cross-sections from DBA/2J, DBA/2J-*Atg4b*^+/−^ and DBA/2J-*Atg4b*^ko^ mice (12 m.o.) ON cross-sectional area was measured and plotted in (**D**). Note the reduction in ON cross-sectional area and ON survival in DBA/2J-*Atg4b*^+/−^ and DBA/2J-*Atg4b*^ko^ mice. **E** Total axon number counts. Data are the means ± SD. **p < 0.01, ANOVA with Bonferroni post hoc test (DBA/2J *n* = 26, DBA/2J-*Atg4b*^+/−^ *n* = 14, DBA/2J-*Atg4b*^ko^ *n* = 32). ns: no significant; arrowheads: multi-laminar myelin sheath; arrows: gliosis.

We next assessed the effect of ATG4B deficiency on ON degeneration. First, we measured the cross-sectional area in the myelinated segment of the ON. ON enlargement with aging has been linked to axon pathology in DBA/2J mice [30]. Interestingly, DBA/2J-*Atg4b*^ko^ mice displayed a significantly smaller ON nerve area compared to DBA/2J littermates (Fig. 4A, D). Higher magnification (Fig. 4B) and ultrastructural images (Fig. 4C) showed, as expected, degenerating axon profiles in the DBA/2J, characterized by multi-laminar myelin sheath (Fig. 4C, *arrowheads*), as well as extensive gliosis (Fig. 4C, *arrows*). Strikingly, no apparent signs of ON degeneration were observed in DBA/2J-*Atg4b*^ko^ mice (Fig. 4B, C). Axon profiles were organized and smaller in size. Some axon enlargement was noted with *Atg4b* heterozygosity. Axon count showed a statistically significant increase in the number of axons in DBA/2J-*Atg4b*^+/−^ and DBA/2J-*Atg4b*^ko^ compared to DBA/2J mice (Fig. 4E).

### Outflow pathway tissue function in *Atg4b*^ko^ mice

**Fig. 5:**
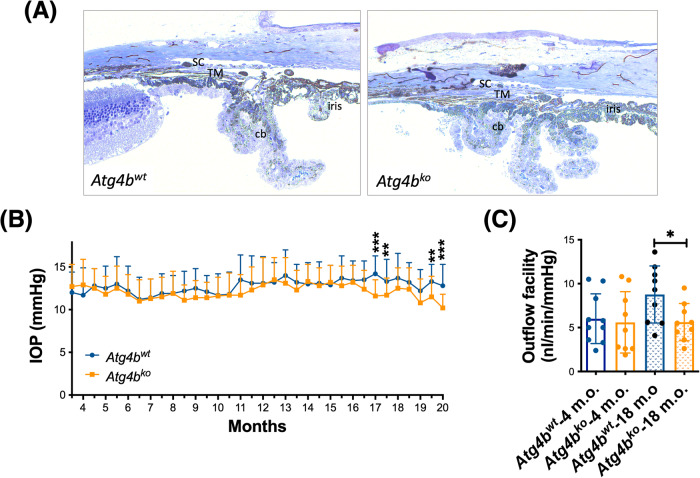
Outflow pathway tissue function in autophagy deficient *Atg4b*^ko^ mice. **A** Toluidine blue-stained histological sections of the iridocorneal region. No morphological abnormalities with *Atg4b* deficiency were noted. Images are representative of 6 animals per group. TM trabecular meshwork, SC Schlemm’s canal, cb ciliary body. **B** Mean IOP (mm Hg) monitored over time in *Atg4b*^wt^ and *Atg4b*^ko^ mice. **p < 0.01, ***p < 0.001, *t* test (*Atg4b*^wt^ *n* = 20, *Atg4b*^ko^ *n* = 21). **C** Ex-vivo outflow facility measures in young (4 m.o) and old (18 m.o.) *Atg4b*^wt^ and *Atg4b*^ko^ mice. *p < 0.05, ANOVA with Bonferroni post hoc test (4 m.o. *Atg4b*^wt^ & *Atg4b*^ko^ *n* = 11; 18 m.o. *Atg4b*^wt^ & *Atg4b*^ko^ *n* = 9).

Our results so far strongly indicate that loss of ATG4B protects against IOP elevation and RGC/ON degeneration. We wanted to further investigate the effect of autophagy deficiency in IOP homeostasis. For this, we characterized the aqueous humor (AH) dynamics and angle structure in *Atg4b*^ko^ mice (C57BL/6J strain). These mice display lower basal levels of autophagy and - as stated earlier - are the only autophagy-deficient mice that are not embryonically lethal [24]. Absence of ATG4B protein levels and higher pro-LC3 in the retina and iridocorneal region were confirmed (Supplementary Fig. 3A). In contrast to DBA/2J-*Atg4b*^ko^, eye cups from *Atg4b*^ko^ mice developed normally and no apparent gross morphological abnormalities were noted in the retina of *Atg4b*^ko^ compared to littermate control mice (Supplementary Fig. 3B, C). ATG4 deficiency did not cause Brn3a^+^ RGC death (Supplementary Fig. 3D, E), and no features of ON degeneration were noted (Supplementary Fig. 3F), although a slight decrease in axon count was found (Supplementary Fig. 3G). In the anterior segment of the eye, the outflow pathway fully develops normally with an open angle and the presence of the TM and SC (Fig. 5A). IOP measurements showed no differences in IOP between *Atg4b*^ko^ and wt in young mice; however, a gradual decline in IOP with aging was observed in *Atg4b*^ko^ mice starting at 16 m.o. of age (Fig. 5B). We evaluated outflow facility in ex-vivo enucleated eyes (4 m.o and 18 m.o.). *Atg4b*^wt^ mice showed a statistically significant increase in outflow facility with aging (*Atg4b*^wt^ 4 mo, 6.01 ± 2.8 nl/min/mmHg, *n* = 11; *Atg4b*^wt^ 18 mo, 8.767 ± 3.2 nl/min/mmHg, *n* = 9). Interestingly, this was not observed in autophagy-deficient mice (*Atg4b*^ko^ 4 mo, 5.6 ± 3.4 nl/min/mmHg, *n* = 11; *Atg4b*^ko^ 18 mo, 5.62 ± 2.1 nl/min/mmHg, *n* = 9) (Fig. 5C). Together, this data strongly indicates a role of autophagy in AH homeostasis with aging.

### ATGB4 deficiency protects against IOP elevation in the TGF-beta ocular hypertensive model

**Fig. 6:**
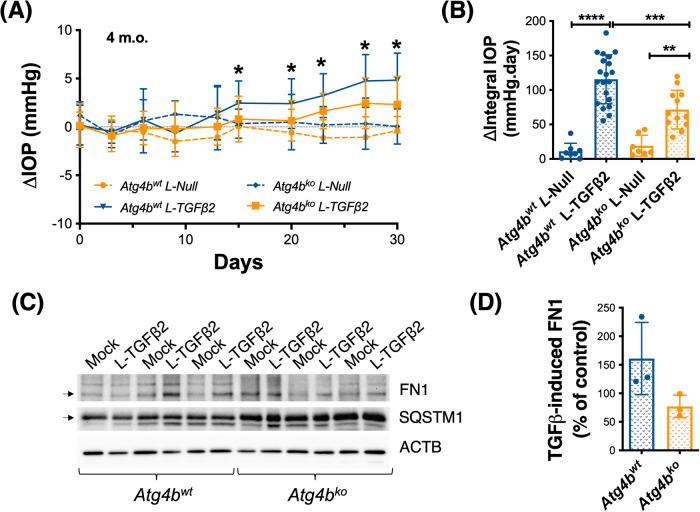
*Atg4b*^ko^ deficiency protects against TGFβ2-induced IOP elevation and fibrosis. *Atg4b*^ko^ and *Atg4b*^wt^ mice (4 m.o.) were intravitreally injected in the right eye with L-TGFβ2 or L-Null virus (2 × 10^6^ TU/eye, 2 μL bolus). The contralateral eye remained non-infected. Nocturnal IOP was monitored twice per week. **A** Mean ΔIOP (mm Hg) calculated between the injected and the mock fellow eye monitored over time. IOP exposure was calculated and plotted on (**B**). *p < 0.05, **p < 0.01, ***p < 0.001, ANOVA with Bonferroni post hoc test (*Atg4b*^*wt*^ L-Null *n* = 8, *Atg4b*^*wt*^ L-TGFβ2 *n* = 21*; Atg4b*^*ko*^ L-Null *n* = 8, *Atg4b*^*ko*^ L-TGFβ2 *n* = 12). **C** WB quantification of fibronectin (FN1) and SQSTM1 protein expression levels in dissected iridocorneal region. ACTB was used for normalization. **D** Quantification of TGFβ2-induced FN1 protein levels from the densitometric analysis of the bands.

The AH from glaucoma patients contains increased TGFβ2 levels [31, 32], which are thought to contribute to the fibrotic phenotype and extracellular matrix deposition reported in the glaucomatous outflow pathway [33, 34]. More, experimental overexpression of TGFβ2 or its downstream effector protein CTGF in the outflow pathway elevates IOP in perfused eyes, rats and mice [35, 36]. A previous study from our lab showed that autophagy deficiency decreases the expression of fibrotic markers and inhibited TGFβ2-induced fibrosis in human TM cells in vitro [37]. We wonder whether ATG4B deficiency could also protect against IOP elevation by TGFβ2. For this, we intravitreally injected *Atg4b*^ko^ and littermates *Atg4b*^wt^ mice (4 m.o.) with a recombinant lentivirus expressing either constitutively active TGFβ2 (L- TGFβ2, 2 × 10^6^ TU/eye, 2 μL bolus) or control null virus (L-Null) [38, 39]. Nocturnal IOP was monitored twice per week, and ΔIOP between the injected and the mock fellow eye was calculated (Fig. 6A). IOP exposure is shown in Fig. 6B. As reported by others, lentiviral delivery of active TGFβ2 gradually elevated IOP, starting at ~12 days post injection and remained elevated throughout the duration of the experiment (Fig. 6A). No IOP elevation was observed in eyes transduced with L-Null virus. Intriguingly, the TGFβ2-induced elevation in IOP was significantly reduced in *Atg4b*^ko^ mice compare to *Atg4b*^wt^ (Fig. 6B, *Atg4b*^*wt*^ L-TGFβ2: 115.6 ± 35.5 mmHg, *n* = 20 vs *Atg4b*^*ko*^ L-TGFβ2: 71.5 ± 28 mmHg, *n* = 12, *p* < 0.0001). No signs of inflammation or gross morphological changes were observed in the outflow pathway region (Supplementary Fig. 4A). We quantified by WB the protein levels of fibronectin (FN1). As seen in Fig. 6C, *Atg4b*^ko^ mice displayed lower TGFβ2-induced FN1 expression (*Atg4b*^*wt*^ L-TGFβ2: 161 ± 63.3%, *n* = 3 vs *Atg4b*^*ko*^ L-TGFβ2: 77 ± 19.7%, *n* = 3, *p* = 0.09), in agreement to our prior findings in vitro [37]. The higher SQSTM1 protein levels confirmed autophagy deficiency in *Atg4b*^*ko*^ mice. Autophagy deficiency did not prevent lentiviral transduction or expression of exogenous TGFβ2 in TM cells (Supplementary Fig. 4B).

## Discussion

Here we have investigated the effect of autophagy deficiency on IOP elevation and RGC degeneration in two different mouse models of glaucomatous chronic IOP elevation: DBA/2J and TGFβ2-induced ocular hypertensive mouse models. Experiments were conducted in *Atg4b*^ko^ and DBA/2J-*Atg4b*^ko^ mice; the later generated in our laboratory via CRISPR/Cas9 technology. Our data strongly indicates that autophagy deficiency protects against glaucomatous IOP elevation, RGC death and ON atrophy. These results are consistent with previous studies from our laboratory, which suggested a pathogenic role of autophagy activation in glaucoma [12, 15].

### Autophagy deficiency and IOP

A major finding in our studies is the impact of autophagy deficiency on the development of glaucomatous IOP elevation. In contrast to DBA/2J, DBA/2J-*Atg4b*^ko^ mice did not develop ocular hypertension with aging. Interestingly, heterozygous mice behaved as WT controls, showing gradual elevation in IOP starting at 7 months of age. Absence of glaucomatous IOP in DBA/2J-*Atg4b*^ko^ mice could not be attributed to any gross morphological changes in the outflow pathway angle region resulting from deletion of *Atg4b*. Both, DBA/2J-*Atg4b*^ko^ and DBA/2J-Atg4^+/−^ mice still presented iris and ciliary body atrophy with severe anterior synechia obstructing the angle structures, as observed in the WT [23]. This modulating effect of ATG4B deficiency on IOP was not limited to the glaucomatous DBA/2J model. ATG4B deficiency also prevented the development of glaucomatous IOP elevation in the experimental TGFβ2 ocular hypertensive model. Furthermore, ATG4B knockdown significantly influenced IOP homeostasis with aging. Our data showed a progressive decline in IOP overtime in *Atg4*^ko^ mice, and no increase in outflow facility with aging was observed in *Atg4*^ko^ mice, as seen in WT controls and also reported in [40]. Our data contrast to that by Kasetti et al. [41], in which elevation in IOP was observed in Atg5^fl/fl^ mice intravitreally injected with Ad5.Cre. In addition to the most obvious potential effects resulting for the different experimental models (i.e full germ-line ko vs conditional ko), we cannot exclude the possibility of distinct individual roles played by ATG4B and ATG5. ATG5 is known to pay a role in immune response against viral infection [42–44]. It is plausible that a combination of knocking down Atg5 with adenoviral vectors could amplify the adenoviral inflammatory response. Nevertheless, these data together strongly support a role of autophagy in IOP regulation.

The specific molecular events by which autophagy regulate IOP are not known and will most likely differ between the experimental models. TGFβ2-induced fibrogenesis with subsequent ECM deposition and stiffness are believed to be the culprit of decreased resistance to AH outflow and IOP elevation in primary open angle glaucoma [33, 34]. Previous studies conducted in our laboratory identified TGFβ/SMAD-induced fibrogenesis as one of the top pathways affected in autophagy-deficient human trabecular meshwork (TM) cells [37]. Genetic or pharmacological inhibition of autophagy prevented the upregulation of fibrotic genes, including fibronectin, in cultured TM cells. Excitingly, our results here confirm these findings in vivo suggesting that inhibition of TGFβ2-induced fibrogenesis might be one of the factors by which autophagy deficiency prevents IOP elevation.

The mechanisms of IOP elevation in the DBA/2J mice, or those in pigmentary glaucoma, are still not well understood. It is believed that the blockage of outflow pathway channels by pigment particles and anterior synechia contributes to ocular hypertension (OHT). However, it is worth noting that normotension is frequently observed despite the presence of these structural abnormalities [45, 46]. ATG4B deficiency did not seem to result in any major anatomical abnormality in the iridocorneal region compared to their respective controls. However, at the ultrastructural level, the presence of intracellular membranous structures was observed in the outflow pathway cells of DBA/2J-*Atg4b*^ko^ mice. These were not observed in *Atg4b*^ko^ mice, suggesting a strain-specific strain. Pigment dispersion syndrome in DBA/2J mice results in the release of pigment particles into the outflow pathway, which are phagocytosed by TM cells. Since autophagy, phagocytosis and LC3-associated phagocytosis share common players [47, 48], an impact of autophagy-deficiency on the endolysosomal system is expected. We speculate that these membranous structuresresult from impaired phagocytic activity and/or phagosome maturation in the autophagy-deficient DBA/2J mice. Further investigation is needed to understand how or if this impairment may affect IOP regulation, including assessing corneal thickness, outflow resistance, and aqueous humor production.

### Autophagy deficiency and RGC survival

The first critical observation from our studies is that diminished basal autophagy does not trigger RGC loss. No differences in RGC counts were observed between *Atg4b*^ko^ and littermate WT mice, indicating that autophagy does not play a critical role in RGC survival during development or under physiological conditions. Furthermore, our data strongly demonstrate that ATG4B deficiency prevents glaucomatous RGC death. DBA/2J-*Atg4b*^ko^ mice showed higher total number of RGC, decreased GFAP immunoreactivity and no apoptotic bodies compared to DBA/2J wt mice. Morphologically, ATG4B deficiency ameliorated thinning of the INL and other retina layers characteristic of DBA/2J mice [23, 27]. Although this phenotype could be attributed to the lack of degeneration resulting from the absence of glaucomatous IOP elevation, it is important to note that RGC survival was also evident in DBA/2J-*Atg4*^+/−^, which experience glaucomatous IOP elevation at levels comparable to those observed in their WT littermates. Our results contrast to published studies, which report a protective role of autophagy against RGC death in glaucoma. There are several explanations for such discrepancy. First, most of the studies claiming a protective role of autophagy were conducted in acute models of OHT [20] or axonal insult [19, 49], which do not faithfully mimic glaucoma pathogenesis. Second, with a few exceptions, autophagy was modulated using pharmacological approaches with non-specific drugs (i.e. rapamycin or 3-MA) [21, 50]. Ours is the only study so far that has investigated the impact of autophagy on RGC survival in a model of chronic glaucomatous IOP elevation using genetic approaches.

A role of autophagy in cell death has been well documented, although the mechanisms are not fully characterized [7, 8, 51, 52]. Autophagy might initiate several forms of cell death by selectively degrading molecules that trigger the process; i.e anti-apoptotic or survival factors. Best studied is the cross-regulation between autophagy and apoptosis. Cleavage and inactivation of the autophagy protein Beclin 1 by caspases leads to autophagy inhibition [53–56]. In contrast, autophagy prevents apoptosis by degrading active caspase-8 or activation of Bid by Beclin 1 [10, 52, 53]. RGC death in glaucoma is known to occur through apoptosis [57–59]. Indeed, similar to our findings, BCL2-associated X protein (BAX) deficiency in DBA/2J mice protected against glaucomatous IOP elevation and RGC death [26]. Therefore, it is plausible that autophagy deficiency might impede or limit activation of apoptosis. This is supported by the absence of apoptotic nuclei in DBA/2J-*Atg4b*^ko^ mice and findings from other groups. For example, in a model of chronic IOP elevation by episcleral vein injection in rats, Zhu et al. observed a neuroprotective effect of ghrelin mediated by inhibition of retinal autophagy, RGC apoptosis, and Muller cell gliosis [60]. Moreover, inhibition of miR-708 and miR-335-3p triggered RGC apoptosis through promoting autophagy in a laser photocoagulation OHT mouse model [61]. A time-dependent bi-phasic role of autophagy has been also suggested. Using the occlusion magnetic beads OHT model in rats, Zhang et al. found a dual role of autophagy, promoting RGC apoptosis in the early stages of glaucoma and autophagic cell death in later stages [62]. Regardless the underlying mechanisms, these studies together with our previous [12, 15] and current data strongly support a role of autophagy in glaucomatous RGC death.

### Autophagy deficiency and glaucomatous axonal degeneration

A major strength in our study compared to prior published work, in which autophagy was independently investigated either in RGC soma or ON, is that we have evaluated the effect of autophagy deficiency on both, RGC and ON axons. This is critical since data suggests the co-existence of distinct somal and axonal degeneration pathways in glaucoma [3]. Interestingly, although autophagy deficiency did not impact somatic Brna3a+ cell death during development or under physiological conditions, it slightly reduced the number of ON axons in *Atg4b*^ko^ mice. In contrast, within the context of glaucomatous insult, autophagy deficiency completely prevented axonal degeneration in either DBA/2J-*Atg4*^+/−^ or DBA/2J-*Atg4*^ko^ mice. Autophagy deficiency also rescued ON enlargement in DBA/2J mice [30].

Limited research has been conducted to elucidate the role of autophagy in axonal degeneration associated with glaucoma. In chronic OHT models, elevation in IOP resulted in the intra-axonal accumulation of autophagic figures and autophagosomes markers, which was attributed to defects in autophagy flux [63, 64]. We observed a similar phenotype in GFP-LC3 mice subjected to the episcleral model of OHT [15] and in DBA/2J::GFP-LC3 [12]. In these, expression of the GFP-LC3 transgene caused a dramatic exacerbation of ON degeneration, associated with a higher presence of large autolysosomes within ON axons.

The accumulation of autophagy-related vesicles due to insufficient autophagic clearance or excessive autophagy induction has been observed in degenerating neurites from patients affected with other neurodegenerative diseases, including Alzheimer’s disease, Parkinson’s disease and Huntington’s disease [65–67]. It is thought that such accumulation of autophagosomes leads to axonal transport failure and axon swelling [65]. While regulation of autophagy in RGCs axons has not yet been investigated, emerging studies in neurons indicate that autophagy is compartmentalized, with formation of autophagosomes both at the soma and in the axon [68, 69]. Autophagosomes that are generated at the soma are relatively immobile and clustered at the perinuclear region, where they fused with lysosomes. In contrast, in the axon, autophagosomes are generated at the distal site and along the length of the axon during axonal stress. Following formation, autophagosomes containing cargo material are retrogradely transported to the soma for maturation. Using serial block face scanning electron microscopy, Kleesattel et al., examined the distribution of autophagic vesicles and mitochondria in the proximal and distal ON axons in the DBA/2J mice [70]. They found increased autophagosomes in the glaucomatous ON optic nerve, along with higher number of mitochondria and decreased LAMP1, which suggested reduced mitophagy. However, although anterograde transport was affected, no significant defects in retrograde transport were observed, suggesting the existence of alternative mechanisms promoting the intra-axonal accumulation of autophagic bodies. Regardless the mechanism, we can argue that a reduction of basal and inducible levels in autophagy, as seen in the DBA/2J-*Atg4*^+/−^ or DBA/2J-*Atg4*^ko^ mice, could protect against axonal degeneration by decreasing or preventing the accumulation of autophagic vacuoles.

## Summary

This represents the first study in which the role of autophagy in RGC death and ON neurodegeneration was investigated in murine models of glaucomatous IOP elevation using a genetic approach. Our data supports a role of autophagy in IOP homeostasis, RGC death and ON neurodegeneration.

## Supplementary information

Supplemental Figures

Supp Material Uncropped WB

AJ Checklist

## Data Availability

All data generated or analysed during this study are included in this published article [and its supplementary information files].
