# Supplementary material for: Autophagy deficiency protects against ocular hypertension and neurodegeneration in experimental and spontaneous glaucoma mouse models
Source: Cell Death Dis. 2023 Aug 24;14(8):554. doi: 10.1038/s41419-023-06086-3 (PMC10449899; doi:10.1038/s41419-023-06086-3)
Supplement: Supplementary file 1 — Supplemental Figures [file 41419_2023_6086_MOESM1_ESM.docx]

**Supplemental Material**

**AUTOPHAGY DEFICIENCY PROTECTS AGAINST OCULAR HYPERTENSION AND NEURODEGENERATION IN EXPERIMENTAL AND SPONTANOUS GLAUCOMA MOUSE MODELS**

Angela Dixon^1^, Myoung Sup Shim^1^, April Nettesheim^1^, Aislyn Coyne^1^, Chien-Chia Su^1^, Haiyan Gong^2^, Paloma B. Liton^1,*^

- Supplemental Figure 1
- Supplemental Figure 2
- Supplemental Figure 3
- Supplemental Figure 4

**Supplemental Figure 1**

**
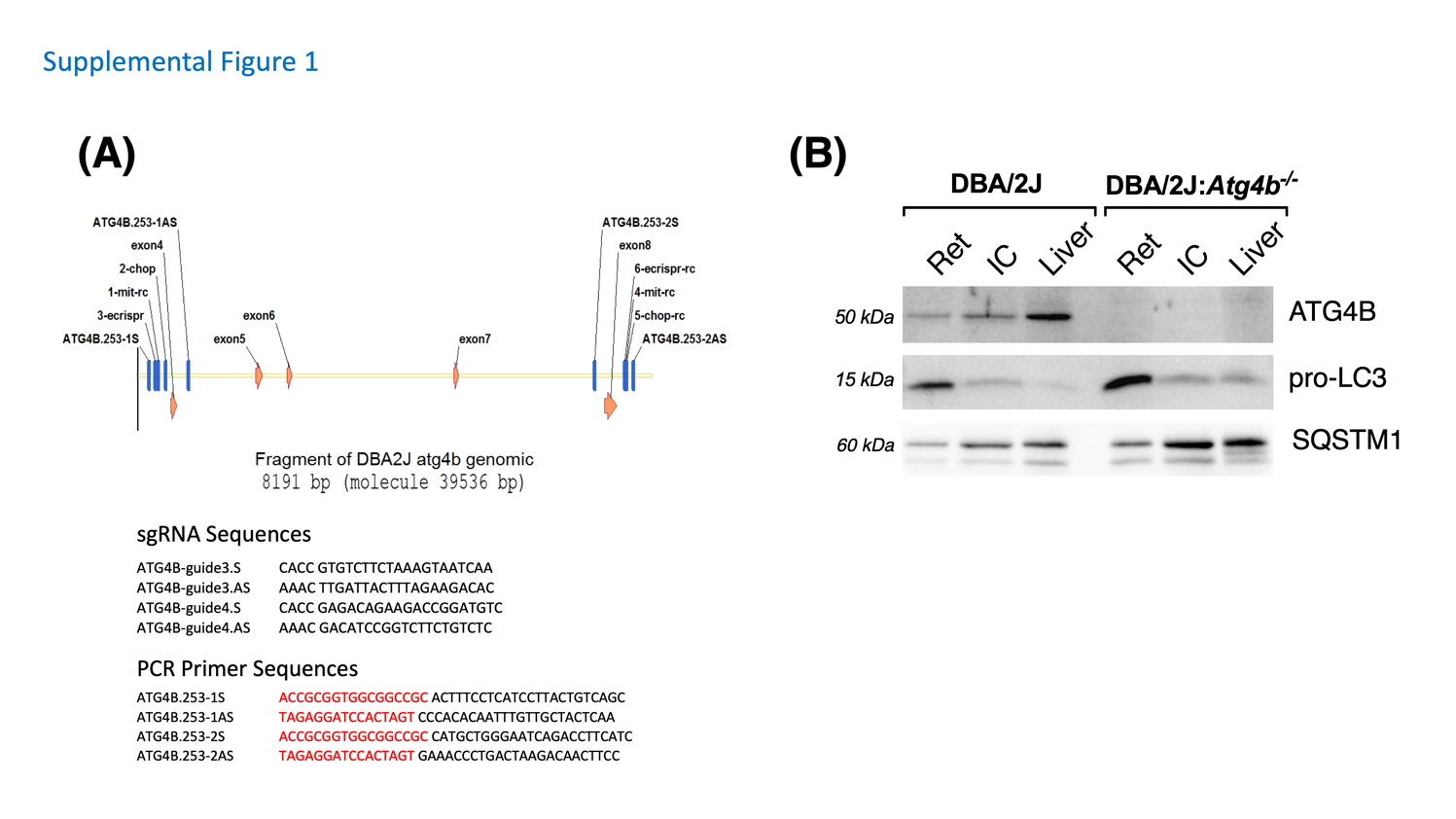
**

**Supplemental Figure 1:** Generation of DBA/2J-*Atg4b*^ko^ mice. **(A)** Genomic location of sgRNAs and PCR primer sequences. Deletion of an ~8 kb region encompassing exons 4-8 in the *Atg4b* gene with sgRNAs 3 and 4 resulted in a frameshift mutation beginning in exon 9 and a premature STOP codon in exon 10. **(B)** WB analysis of dissected retina, iridocorneal region and liver showing the absence of ATG4B and higher pro-LC3 and SQSTSM1 protein levels in the DBA/2J-*Atg4b*^ko^ mice, indicating deficient autophagy. IC: iridocorneal.

**Supplemental Figure 2**


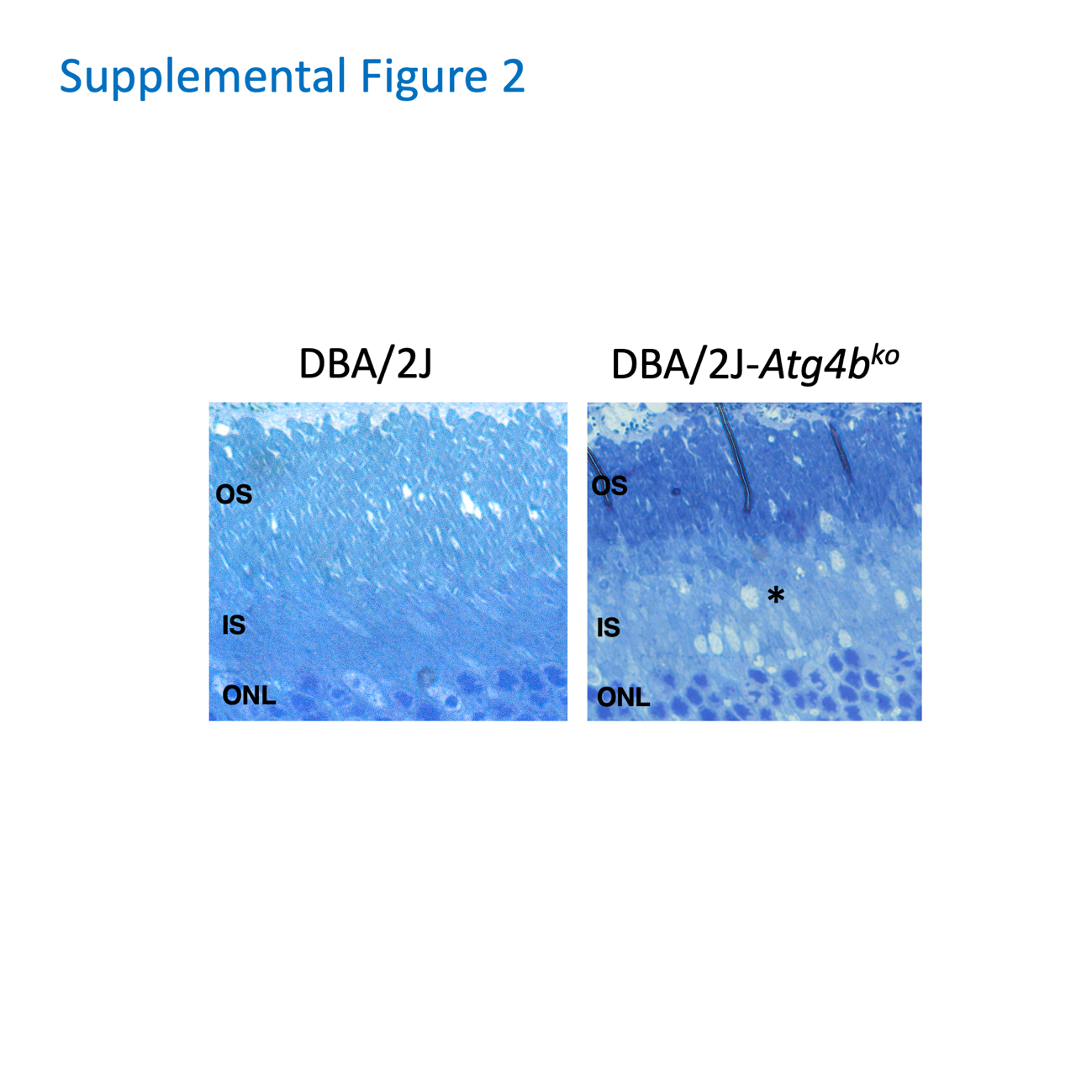


**Supplemental Figure 2:** Higher magnification of cross-sectional histological images showing disorganization of the OS and IS in the DBA/2J-*Atg4b*^ko^ mice. Note that IS in photoreceptors is thicker and display some vacuolization (asterisk). Lamellaes in the OS appear to be shorter and denser compared to WT. Images are representative of at least 6 animals per group. OS: outer segment; IS: inner segment; ONL: outer nuclear layer.

**Supplemental Figure 3**


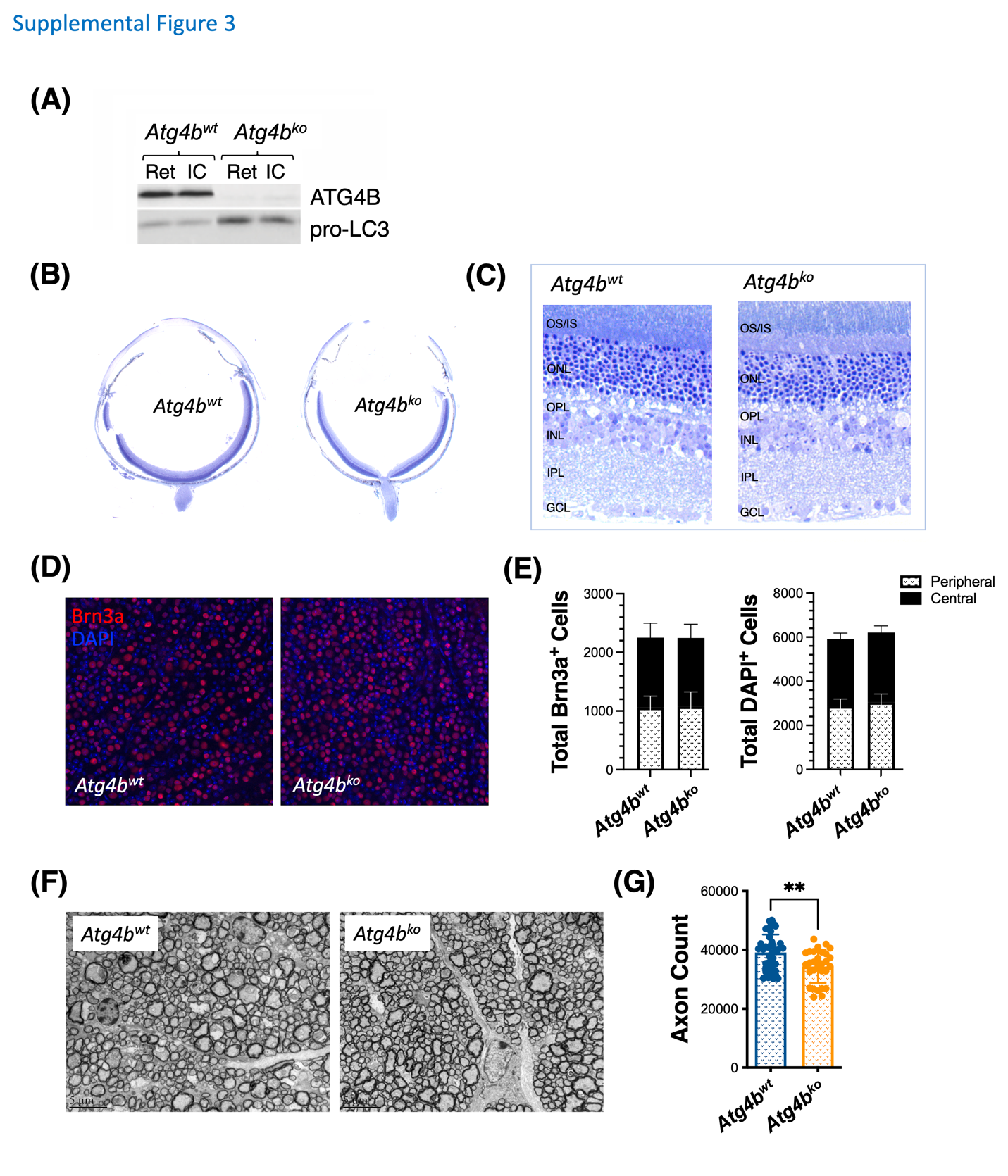


**Supplemental Figure 3:** Morphological characterization of *Atg4b*^ko^ mice. **(A)** WB analysis of dissected retina, iridocorneal region and liver showing the absence of ATG4B and pro-LC3 in the *Atg4b*^ko^ mice. IC: iridocorneal. **(B)** Cross-sectional histological images of eye cups from *Atg4b*^Wt^ and *Atg4b*^ko^ mice (3 m.o.). **(C)** Cross-sectional retinal histological images. No gross morphological differences with *Atg4b* deficiency were found. Images are representative of at least 6 animals per group. OS: outer segment; IS: inner segment; ONL: outer nuclear layer; OPL: outer plexiform layer; INL: inner nuclear layer; IPL: inner plexiform layers; GCL: ganglion cell layer. **(D)** Representative images from whole retinal flat mounts from *Atg4b*^Wt^ and *Atg4b*^ko^ mice (3 m.o.) stained with anti-Brn3a (red fluorescence). Blue represents DAPI nuclear staining. Total Brn3a-positive and DAPI-positive cells in the peripheral and central retina were quantified and plotted in **(E)**. Data are the means ± SD; Tukey test; (*Atg4b*^wt^ n=14, *Atg4b*^ko^ n= 12). **(F)** Electron micrographs of ON cross-sections from *Atg4b*^Wt^ and *Atg4b*^ko^ mice (3 m.o.). Total axon numbers were counted and represented in **(G)**. Data are the means ± SD. **p<0.01, t-test (*Atg4b*^wt^ n=40, *Atg4b*^ko^ n= 23).

**Supplemental Figure 4**


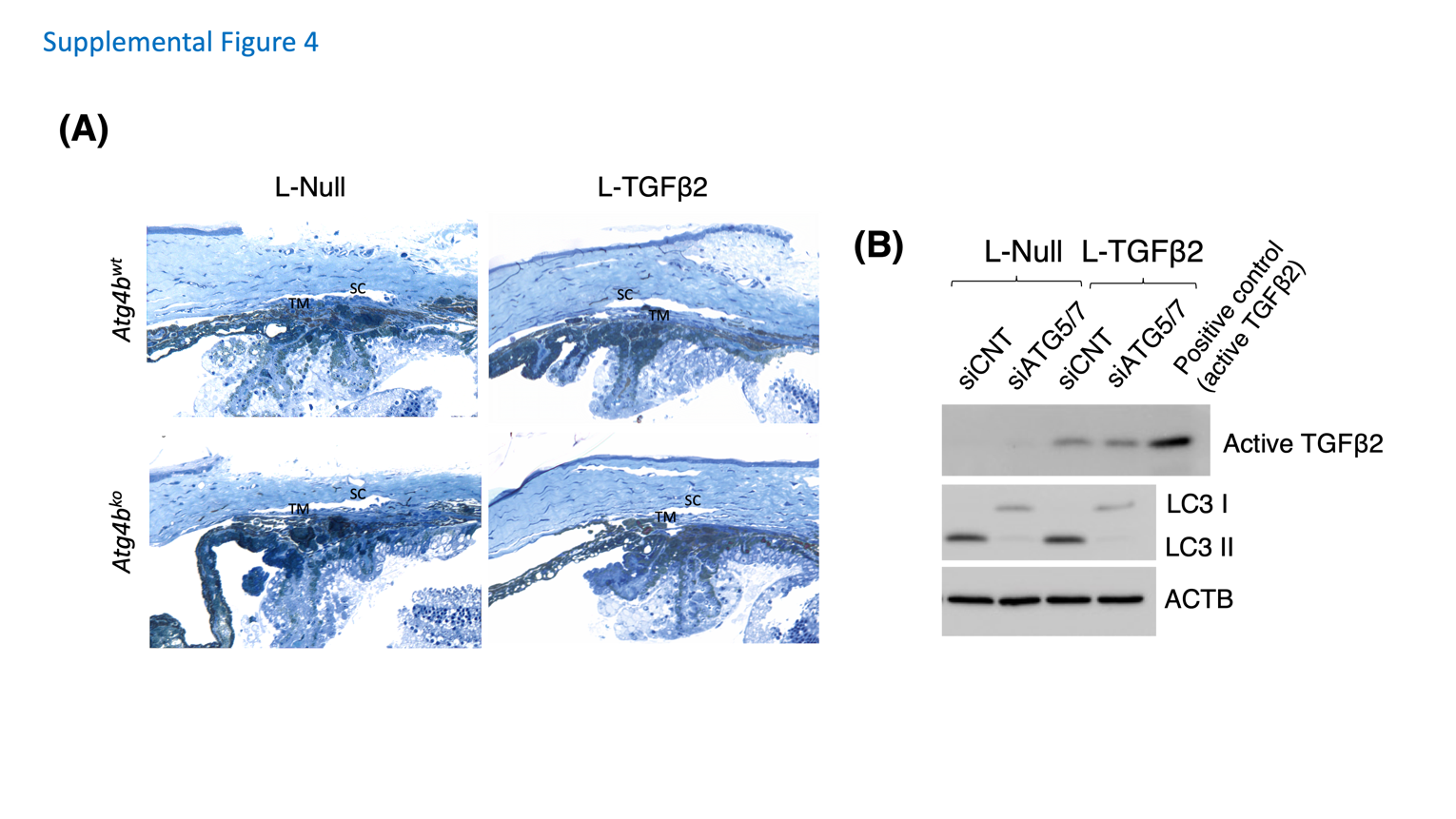


**Supplemental Figure 4: (A)** Toluidine blue-stained histological sections of the iridocorneal region of L-Null and L-TGFβ2-injected eyes. No signs of inflammatory response were noted. Images are representative of 6 animals per group. TM: trabecular meshwork, SC: Schlemm’s canal. **(B)** Primary cultures of human TM cells were transfected with siRNAs targeting Atg5/Atg7 (siAtg5/7) or scrambled siRNA (siCNT) as previously described (37) and transduced with either L-Null or L-TGFβ2 (10 pfu). Protein levels of active TGFβ2 and LC3 were evaluated in the culture media and whole cell lysate, respectively. Autophagy deficiency did not affect expression of L-TGFβ2.
