# Supplementary material for: Autophagy deficiency protects against ocular hypertension and neurodegeneration in experimental and spontaneous glaucoma mouse models
Source: Cell Death Dis. 2023 Aug 24;14(8):554. doi: 10.1038/s41419-023-06086-3 (PMC10449899; doi:10.1038/s41419-023-06086-3)

Supplemental Figure 1

(B)

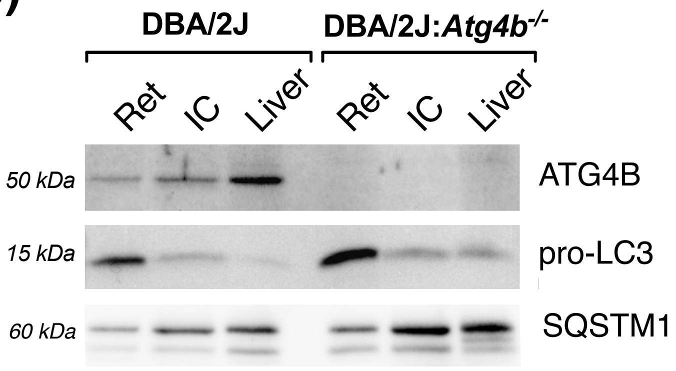

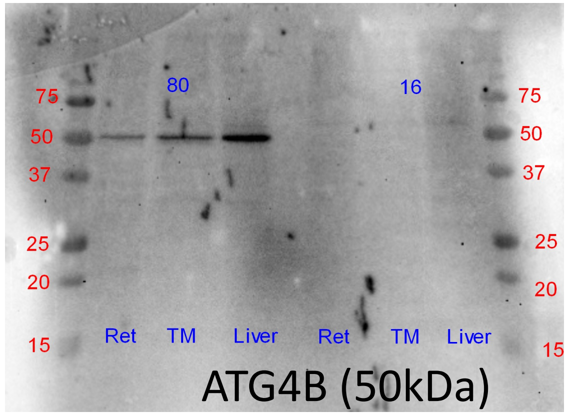

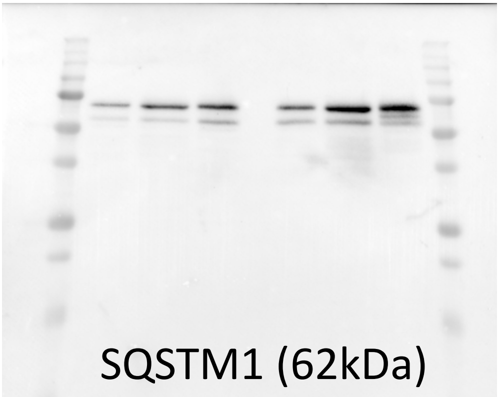

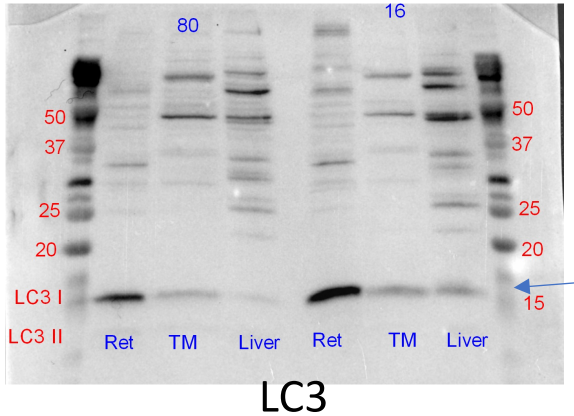

LC3

LC3

(pro-LC3 and LC3-I run around 15-20kDa)

Supplemental Figure 2

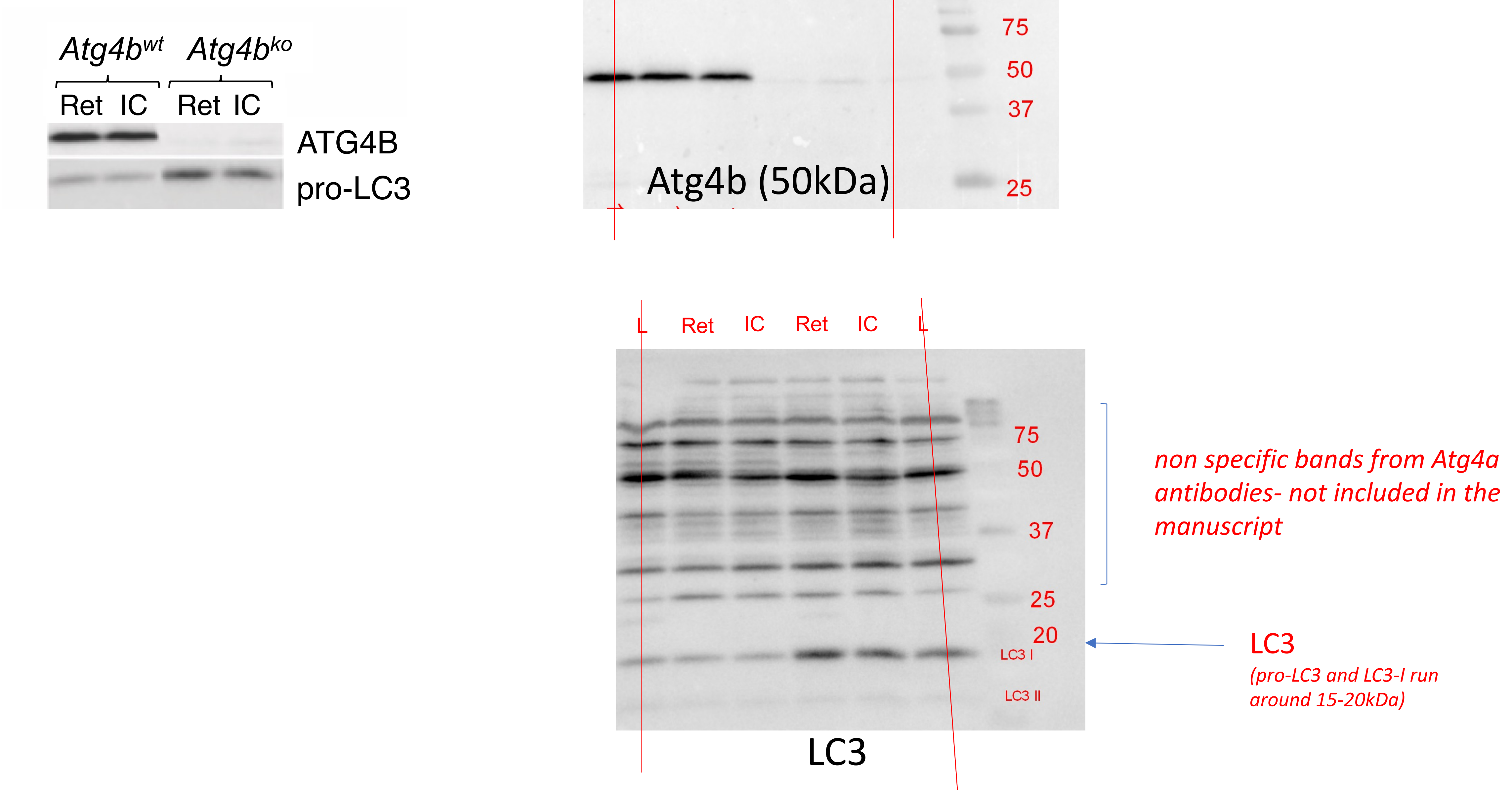

Supplemental Figure 3

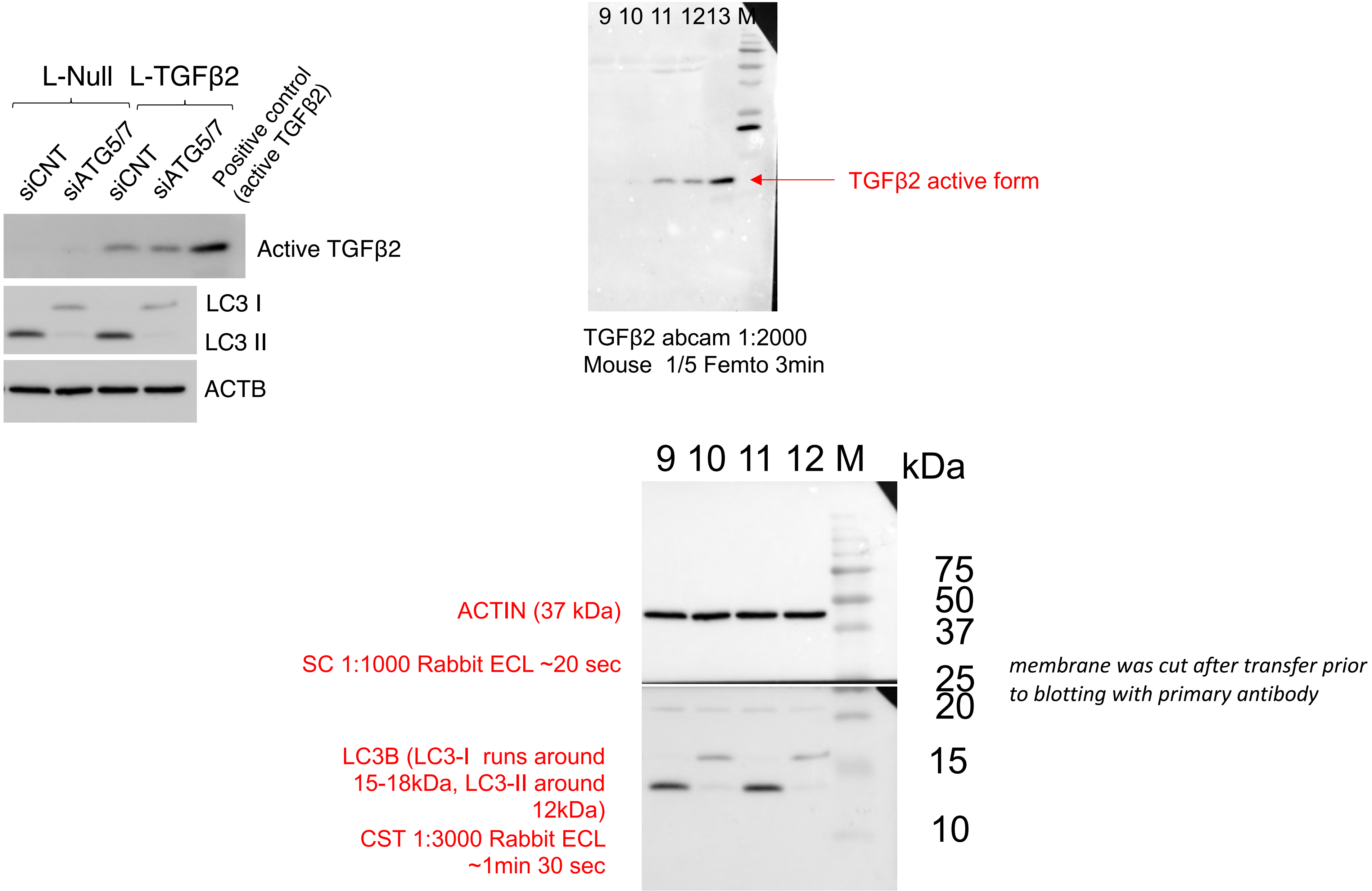

Figure 6

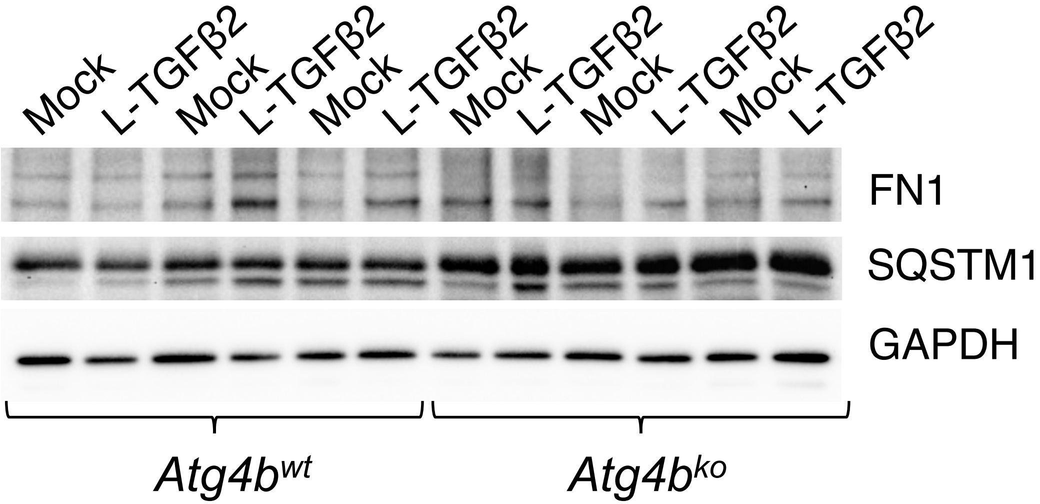

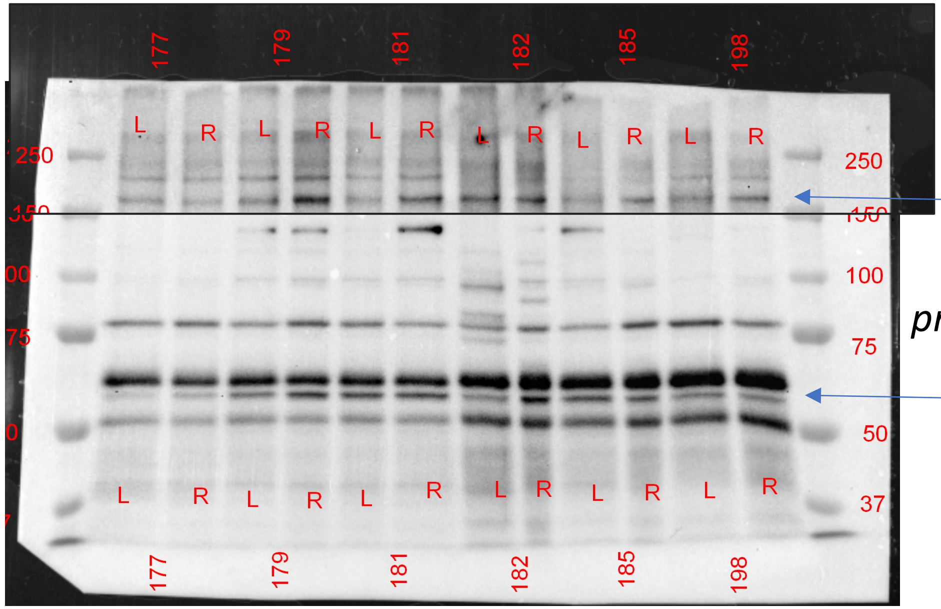

FN1

prior primary ab

SQSTSM1
62kDa

membrane was cut after transfer prior to blotting with primary antibody

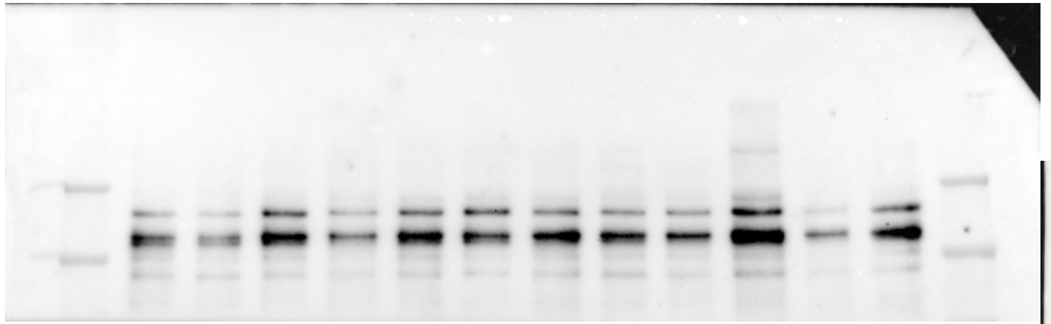

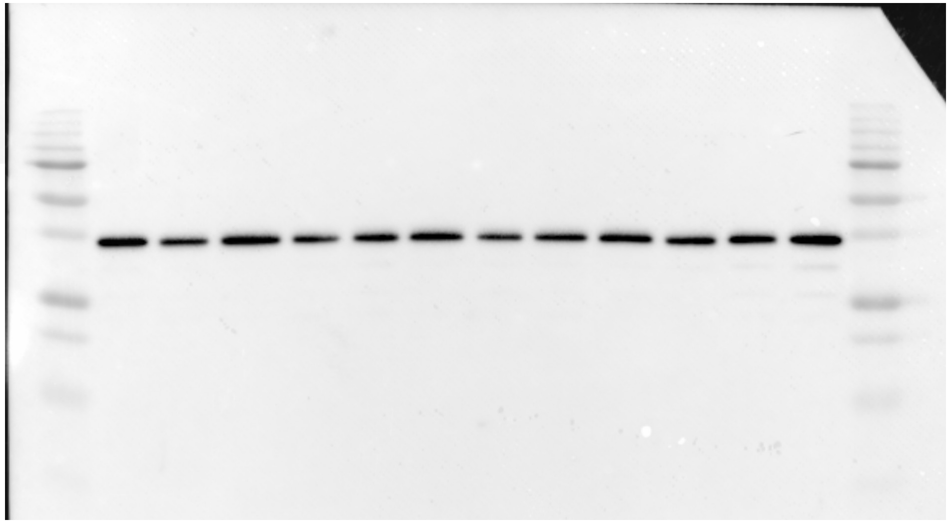

GAPDH
36kDa

Figure 6

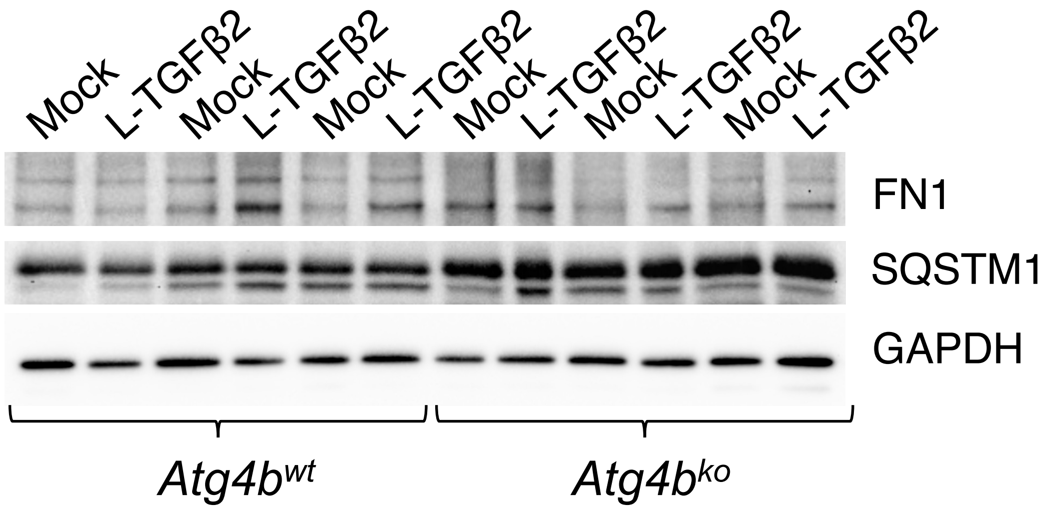

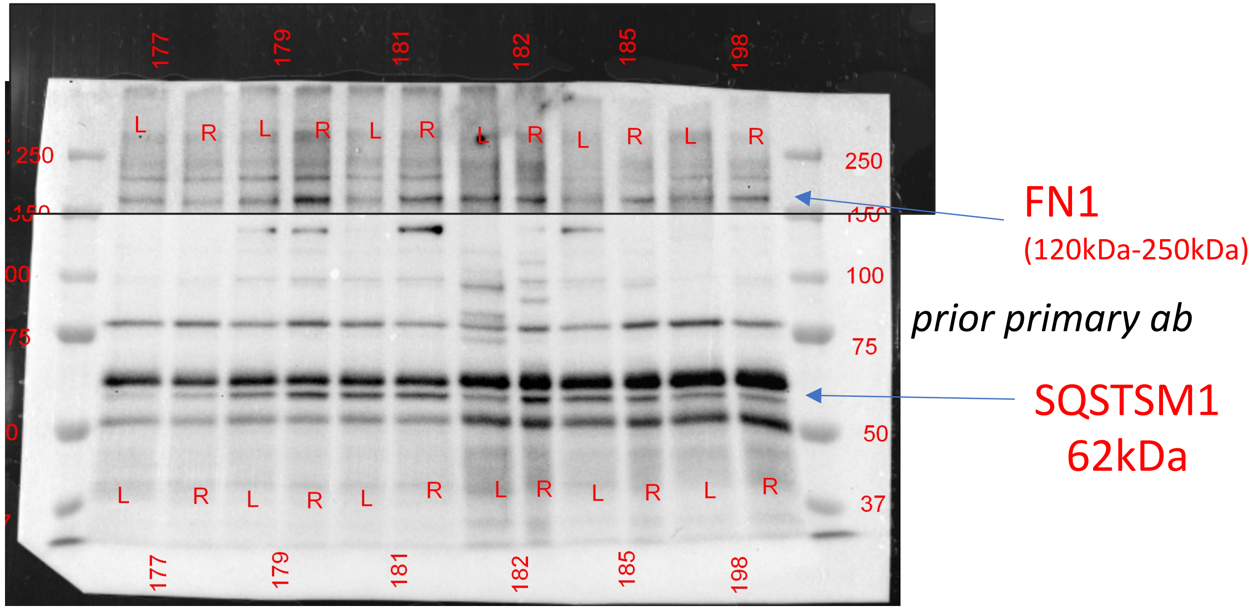

membrane was cut after transfer prior to blotting with primary antibody

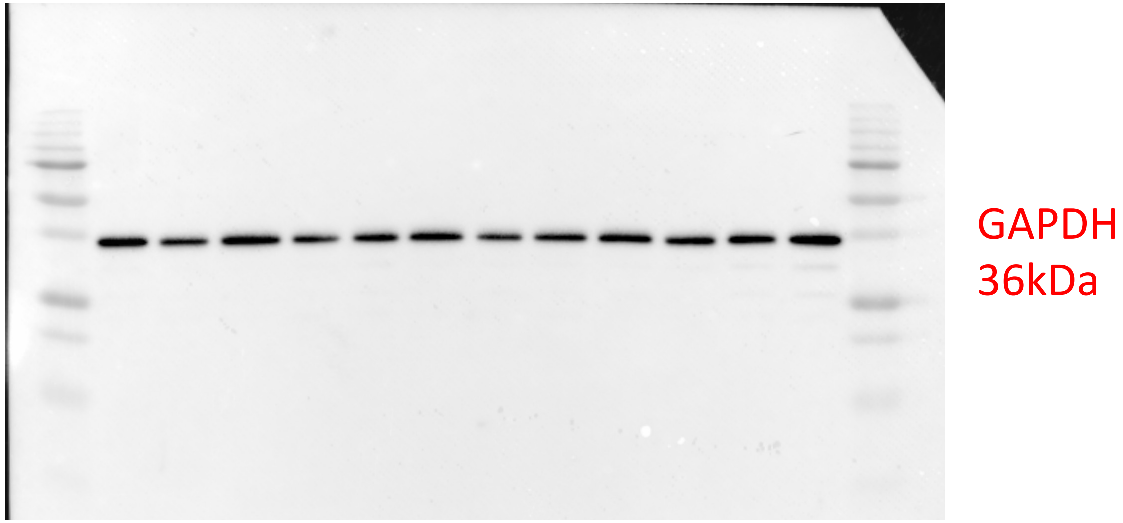

Supplement: Supplementary file 2 — Supp Material Uncropped WB [file 41419_2023_6086_MOESM2_ESM.pdf]
